# Is quality of YouTube content on Bankart lesion and its surgical treatment adequate?

**DOI:** 10.1186/s13018-020-01590-0

**Published:** 2020-02-26

**Authors:** Ahmet Onur Akpolat, Demet Pepele Kurdal

**Affiliations:** Department of Orthopaedics and Traumatology, Fatih Sultan Mehmet Training and Research Hospital, Istanbul, Turkey

**Keywords:** YouTube, Surgical, Treatment, Quality, Bankart

## Abstract

**Background:**

The Internet has developed into a fast and easy to access source of information. The second most popular social media network is YouTube. We aimed to evaluate the accuracy and quality of videos uploaded to YouTube about Bankart lesion without diagnostic or treatment-related criteria.

**Methods:**

Various keywords were searched for on YouTube. Videos were evaluated with the DISCERN and JAMA Benchmark scoring systems by two independent reviewers.

**Results:**

A total of 48 videos were taken into evaluation as a result of the search. The mean view count was 28909.68 ± 30264.3. Mean length of the videos was 313,06 ± 344.65. The average DISCERN score of both reviewers was 2.35 ± 0.91. The average JAMA Benchmark score of both reviewers was 2.11 ± 0.77.

**Conclusion:**

We concluded that the accuracy and reliability of the videos obtained from YouTube by searching for the words Bankart and labrum lesion/injury/treatment are low.

## Introduction

The Internet has developed into a fast and easily accessible source of information [1]. It is estimated that total Internet use between the years 2000 and 2017 has increased by 962.6% and that 51% of the world population has access to the Internet [2]. Rate of social media use between ages 18–29 is about 90% [1–3]. Nowadays, the second most commonly used social media network is YouTube, a global social network translated into 76 different languages, used in 88 countries, with over one billion users [4]. YouTube has become an incredible rapid-growing visual database with over 300 video uploads per minute and more than 100 million hours of video views per day [4]. In 2014, according to a study conducted in the USA, YouTube use was 80% between ages 14 and 29 and 90% between ages 18 and 49 [3, 4].

Although the main purpose of YouTube is entertainment rather than educational purposes, over time, due to patient interest, it has also become a platform for medical information for academicians and colleagues as well as communication with patients. Videos uploaded to YouTube do not pass an editorial process and most do not contain information on authorship or origin. Users are unfamiliar with the accuracy or reliability of the resource. They may also be subject to misleading advertisements.

When we searched for the word “YouTube” on PubMed (March 10, 2019), we encountered close to a thousand results. A majority of the studies were evaluations of the quality of the content obtained from YouTube. This large number of studies suggest that the quality of information obtained from YouTube is controversial [5]. The Internet is also widely used by orthopedic patients to learn information about their disorders [6].

The Bankart lesion is a lesion of the anterior glenoid labrum of the shoulder [7] and is most commonly caused by recurrent dislocation of the shoulder, with an incidence of 1.7%. Bankart lesion is found in 80% of patients with recurrent dislocation of the shoulder [7, 8] and is most common between 18 and 30 years of age [8].

Our study’s objective was to determine the quality of YouTube videos related to the diagnosis and treatment of patients with Bankart lesion, which is most commonly observed in the young population.

## Material and methods

On March 5, 2019, a search containing various keywords was conducted on YouTube (http://www.youtube.com) including “Bankart,” “Bankart lesion,” “Bankart surgery,” and “Bankart Repair,” along with “Labrum,” “Labrum tear,” “Labrum repair,” and “Labrum surgery” due to the fact that patients could easily access their magnetic resonance reports.

**Fig. 1:**
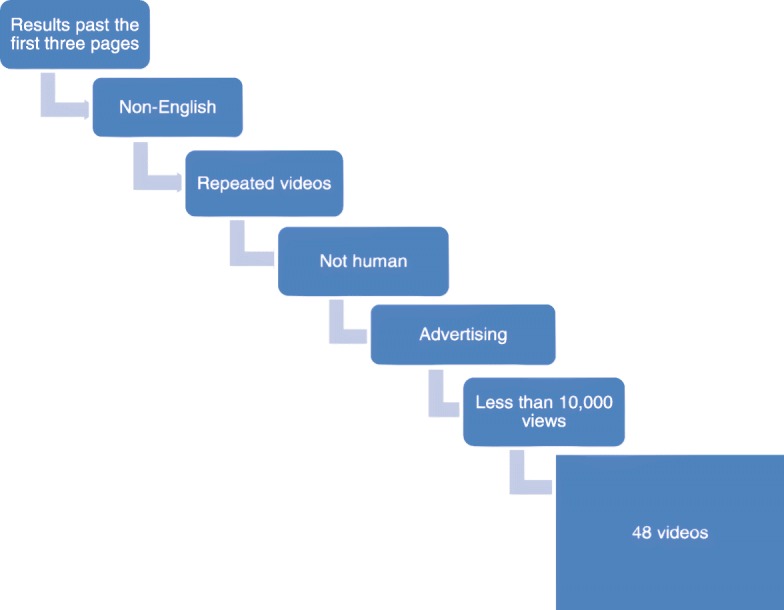
Exclusion criteria

Studies have shown that less than 17% of Internet users view results beyond the first three pages of the search results [9]. Therefore, in our study, we only evaluated the first three pages of the results. The other exclusion criteria included repeated videos, non-English videos, inhumane videos, videos that included advertising content, and videos that were viewed less than 10,000 times (Fig. 1). Videos were divided into groups based on criteria such as type (animation, surgical, cadaver, etc.) and upload year, then evaluated using two different scoring systems (DISCERN, JAMA Benchmark).

DISCERN is a scoring system developed at Oxford and used to evaluate the quality of health care. It is originally made up of 16 questions. A score of 1 to 5 is given for each question. The lower score limit is 6 and the upper score is 80 [10]. Singh et al. modified DISCERN for the evaluation of YouTube. Scoring for clarity, reliability, bias/balance, providing of additional information, and uncertainty criteria were established. A score between 0 and 5 is given for each set of criteria. A higher score represents higher video quality [11].

The JAMA Benchmark evaluates the quality of information obtained from the Internet with four criteria. A score is given for each criterion: Internet uploaders (who or by whom they are made, uploaded, and the credentials of such persons), source (explicitly declaring the copyrights of the references and resources contained in the content), explanation (any sponsorship, advertising, commitment, commercial financing of the website), and validation (including comments and updated dates) [12]. The scores are between 0 and 4, in which a higher score indicates higher video quality.

## Statistical evaluation

Results obtained from the study were statistically analyzed using the IBM SPSS Statistics 22 (IBM SPSS, Turkey) program. When study data was evaluated, the Shapiro-Wilk test was used to assess the relevance of normal distribution of the parameters. Aside from descriptive statistical methods (mean, standard deviation, frequency), in the assessment of quantitative data, the Kruskal-Wallis test was used to compare parameters with and without normal distribution. For the assessment of DISCERN and JAMA Benchmark scores, intraclass correlation (ICC) was calculated to determine the consistency between the reviewers. *P* > 0.05 was considered significant.

## Results

The YouTube search yielded 1,864,743 results. After applying exclusion criteria, 48 videos were taken for evaluation. According to the parametric assessment of the videos, the number of views was between 10,585 and 306,958 and was a mean of 60,604.12 ± 78,366.9. The length of the videos was between 4 and 3363 s and was a mean of 424.43 ± 566.68 (Table 1). There were 6 videos from 1 clinic, and 3 videos each from 4 different clinics.

**Table 1:** Evaluation of the study parameters

		Min–max	Mean ± SD
Number of views		10,585–306,958	60,604.12 ± 78,366.9
Length (s)		14–3363	424.43 ± 566.68
Upload year	2007	3	6.25
	2008	5	10.41
	2009	5	10.41
	2010	8	16.66
	2011	4	8.33
	2012	7	14.58
	2013	6	12.5
	2014	3	6.25
	2015	3	6.25
	2016	3	6.25
	2017	1	2.08
Video type	Animation	13	27.08
	Surgical	32	66.6
	Cadaver	3	6.25

The year with the highest number of uploads was 2010 with an upload rate of 16.66%, while the least number of videos were uploaded in 2017 with an upload rate of 2.08%. Of the evaluated videos, 32 (66.66%) were surgical, 13 (27.08%) were animations, and 3 (6.25%) were cadaver videos. Four of the 5 most viewed videos were animations, and 1 was a surgical video. There were 6 videos from 1 clinic, and 3 videos each from 4 different clinics.

### Modified DISCERN

The average DISCERN score by reviewer 1 was 2.35 ± 0.98. The average DISCERN score by reviewer 2 was 2.35 ± 0.95. The average score of both reviewers was 2.35 ± 0.91 (Table 1). The consistency of the DISCERN score between both reviewers was 78.6%, which was statistically significant (*p* = 0.000; *p* < 0.05) (Table 2).

**Table 2:** Evaluation of JAMA Benchmark and DISCERN scoring

	Min–Max	Mean ± SD
DISCERN reviewer 1	1-4	2.35 ± 0.98
DISCERN reviewer 2	1-4	2.35 ± 0.95
JAMA reviewer 1	1-3	2.1 ± 0.79
JAMA reviewer 2	1-3	2.13 ± 0.76
DISCERN score	1-4	2.35 ± 0.91
JAMA score	1-3	2.11 ± 0.77

### JAMA Benchmark

The average JAMA Benchmark score by reviewer 1 was 2.1 ± 0.79. The average JAMA Benchmark score by reviewer 2 was 2.13 ± 0.76. The average score of both reviewers was 2.11 ± 0.77 (Table 1). The consistency of the JAMA Benchmark scores of both reviewers was 97.3%, which was statistically significant (*p* = 0.000; *p* < 0.05) (Table 3).

**Table 3:** Consistency levels between DISCERN and JAMA Benchmark scores of the reviewers

	ICC	95% CI		*p*
DISCERN	0.786	0.603	0.891	0.000*
JAMA Benchmark	0.973	0.945	0.987	0.000*

*ICC* intraclass correlation coefficient

*p < 0.05

There was no statistically significant difference in DISCERN or JAMA Benchmark scores according to video type (*p* > 0.05) (Table 4)

**Table 4:** Evaluation of DISCERN and JAMA Benchmark scores according to video type

Video type	DISCERN		JAMA Benchmark	
	Mean ± SS (median)		Mean ± SS (median)	
Animation	2.64 ± 0.69 (2.5)		2 ± 0.58 (2)	
Surgical	2.38 ± 0.95 (2.5)		2.19 ± 0.81 (2)	
Cadaver	1.5 ± 0.87 (1)		1.83 ± 1.04 (1.5)	
*p*	*0.241*		*0.645*	

Kruskal-Wallis test

## Discussion

The main reason we presented this hypothesis in our study was the significant increase in the number of patients who had searched the Internet and applied to our outpatient clinic. Image search is a common type of search method. YouTube is a social network of high interest due to its ease of access to information [13]. Healthy sources of information on the Internet may increase patient satisfaction and compliance with treatment [14, 15]. However, the accuracy and quality of the information obtained by patients cannot be evaluated.

In our study, we found that the videos obtained from a YouTube search containing the words “Bankart lesion/injury/treatment” and “Labrum tear/repair/surgery” were of poor quality. It is known that low quality medical information obtained from YouTube has a negative effect on doctor-patient relationship [16].

When we scanned the literature, we encountered a large number of publications on evaluating video quality of different branches and diseases and all of them concluded that the accuracy of the information and quality of the videos were poor. The results of our study were consistent with the results of those studies [17–25].

Four of the top five most viewed videos were animations. The studies we encountered in the literature also had high view rates for animated videos [25]. They attributed this to the fact that animated videos were visually simple and easy to understand [22].

The most common video type was surgical videos, but had the lowest view rate, similar to other studies. Previous studies suggested that this lower view rate was due to the content being too complex for viewers without medical education and were visually unappealing [25, 26].

Many of the videos were from the same or similar clinics. According to the literature, the mutual opinion was that the surgeon’s desire to raise awareness of themselves and their clinics was the main cause of this situation [25–27].

Although both tests used in the study have been widely used in many publications and tested for reliability, we reevaluated the consistency of both tests within the groups. Obtained data showed high intra-group compliance [25–28].

We found that video quality was poor regardless of video type. Studies in the literature have yielded similar results [25–29].

There are various studies in the literature on orthopedic disorders and their surgical treatments, including distal radius fracture [20], carpal tunnel syndrome [21], pediatric orthopedics [19], cervical disk herniation [22], spinal stenosis treatment [29], and knee arthroscopy and injuries [18]. In these studies, popular search engines (Google/Yahoo/Yandex) were investigated instead of YouTube. In conclusion, it was found that information acquired from the Internet was insufficient and sometimes inaccurate [19–22, 25, 29]. Another study reported that significant correlations were observed between the video’s usefulness and the uploaded source, as well as between the video’s usefulness and viewers’ preferences, such as the number of views, views per day, and number of likes [30].

There are limited orthopedic studies that evaluate the accuracy and quality of YouTube content. Staunton et al. evaluated results of a YouTube search regarding scoliosis using JAMA Benchmark scoring and found that the information was of poor quality [26]. JAMA Benchmark and DISCERN scoring were also used in studies on femoroacetabular impingement syndrome [27], hip arthritis [28], and anterior cruciate ligament (ACL) injury and reconstruction [25], as in our study. The results of the aforementioned studies were similar to ours, in that the information acquired from YouTube was of insufficient-low quality.

Some studies state that the information accessed from YouTube is insufficient and that doctors should present an alternative to prevent patients from misinformation [12]. YouTube videos could be used as learning sources for shoulder physical examinations after the application of appropriate filtering processes, such as review of the upload source and viewers’ preferences [30].

Our study had some limitations. These were as follows: the search and results were momentary, and factors such as YouTube’s coding system, the search history of the IP address of the computer, and localization having an unknown effect on the search results.

## Conclusions

Medicine is a field, due to its nature, in constant communication with people, regardless of branch. We believe that the quality of information obtained from a platform that we have no intervention over is significant. It should not be forgotten that providing patients with an accurate, quality flow of information will reduce the need for an additional need of information during treatment. However, no matter what, it may be difficult to suppress the curiosity and need to research due to human nature.

## Data Availability

The datasets generated and/or analyzed during the current study are available from the corresponding author on reasonable request
